# Investigation of complex nonlinear dynamic behaviors observed in a simplified driveline system with multistage clutch dampers

**DOI:** 10.1038/s41598-022-13833-7

**Published:** 2022-06-14

**Authors:** Jong-Yun Yoon, Byeongil Kim

**Affiliations:** 1 Department of Mechatronics Engineering, Incheon National University, Incheon, 22012 Republic of Korea; 2 School of Mechanical Engineering, Yeungnam University, Gyeongsan, 38541 Republic of Korea

**Keywords:** Engineering, Mechanical engineering

## Abstract

The results of the harmonic balance method (HBM) for a nonlinear system generally show nonlinear response curves with primary, super-, and sub-harmonic resonances. In addition, the stability conditions can be examined by employing Hill’s method. However, it is difficult to understand the practical dynamic behaviors with only their stability conditions, especially with respect to unstable regimes. Thus, the main goal of this study is to suggest mathematical and numerical approaches to determine the complex dynamic behaviors regarding the bifurcation characteristics. To analyze the bifurcation phenomena, the HBM is first implemented utilizing Hill’s method where various local unstable areas are found. Second, the bifurcation points are determined by tracking the stability variational locations on the arc-length continuation scheme. Then, their points are defined for various bifurcation types. Finally, the real parts of the eigenvalues are analyzed to examine the practical dynamic behaviors, specifically in the unstable regimes, which reflect the relevance of various bifurcation types on the real part of eigenvalues. The methods employed in this study successfully explain the basic ways to examine the bifurcation phenomena when the HBM is implemented. Thus, this study suggests fundamental method to understand the bifurcation phenomena using only the HBM with Hill’s method.

## Introduction

The harmonic balance method (HBM) is efficiently used to investigate nonlinear dynamic behaviors in a torsional system induced by piecewise-type nonlinearities under sinusoidal input conditions, especially to examine multiple numbers of periodic responses^1–16^. In addition, Hill’s method can be employed to reveal the stability conditions, while the system analysis is conducted under the frequency up- or down-sweeping conditions^5,6,17^. However, the incremental HBM employed in this study has some limitations in examining complex dynamic behaviors because the basic mathematical model is constructed based on integer valued harmonic terms^5,6^. In addition, the employed Hill’s method can only project the information of the stability conditions. Thus, it is difficult to recognize the practical dynamic behaviors that occur in the physical system, especially regarding the unstable regimes determined by Hill’s method.

Many studies have been conducted to resolve these problems. For example, nonlinear output frequency response functions (NOFRFs) as a type of HBM have been suggested to examine the nonlinear motions of the Duffing oscillator^10^. In this study, NOFRFs in strong nonlinear equations were implemented by employing the Volterra series to extend the classic FRF to nonlinear cases. The incremental harmonic (IHB) method was used to investigate the limit cycle oscillation of a two-dimensional airfoil with parameter variability in an incompressible flow^11^. Here, the strong nonlinear cubic stiffness subject to non-probability was estimated using the IHB method. In addition, an excitation perturbation method to trigger a sub-harmonic resonance has been suggested^18^. To capture the sub-harmonic effects, we artificially modified the input conditions with respect to the relevant sub-harmonic input terms. In addition, various prior studies have been conducted to examine the nonlinear dynamic responses in a torsional system with clearance-type nonlinearities by employing a multiterm HBM^19–24^.

Based on prior studies of various nonlinear analysis problems, this study suggests a method to investigate the bifurcation characteristics using both the HBM and Hill’s method. Thus, the specific objectives of this study are as follows: First is to suggest a method to determine the bifurcation point (BP) of the HBM solutions with respect to the arc-length vector. Here, the BPs are defined along with the saddle node relevance by estimating the angles of the arc-length from the unit vector. Second is to investigate the relationship of the eigenvalue real parts on the bifurcation characteristics. In general, the unstable regimes—except for the saddle-node—are closely related to the various bifurcation types, such as period-doubling, quasi-periodic, and chaotic responses, which can be anticipated along with the positive valued eigenvalue real parts. To achieve these goals, we focus on a torsional system with 1-DOF induced by piecewise-type nonlinearities^6,25^. In addition, the damping value will be given with a specific one, since the nonlinear effects at the super- and sub-harmonic regimes are generally reduced or faded away along with increasing the damping^6^.

## Problem formulation and mathematical model

### Practical system and its schematic

**Figure 1:**
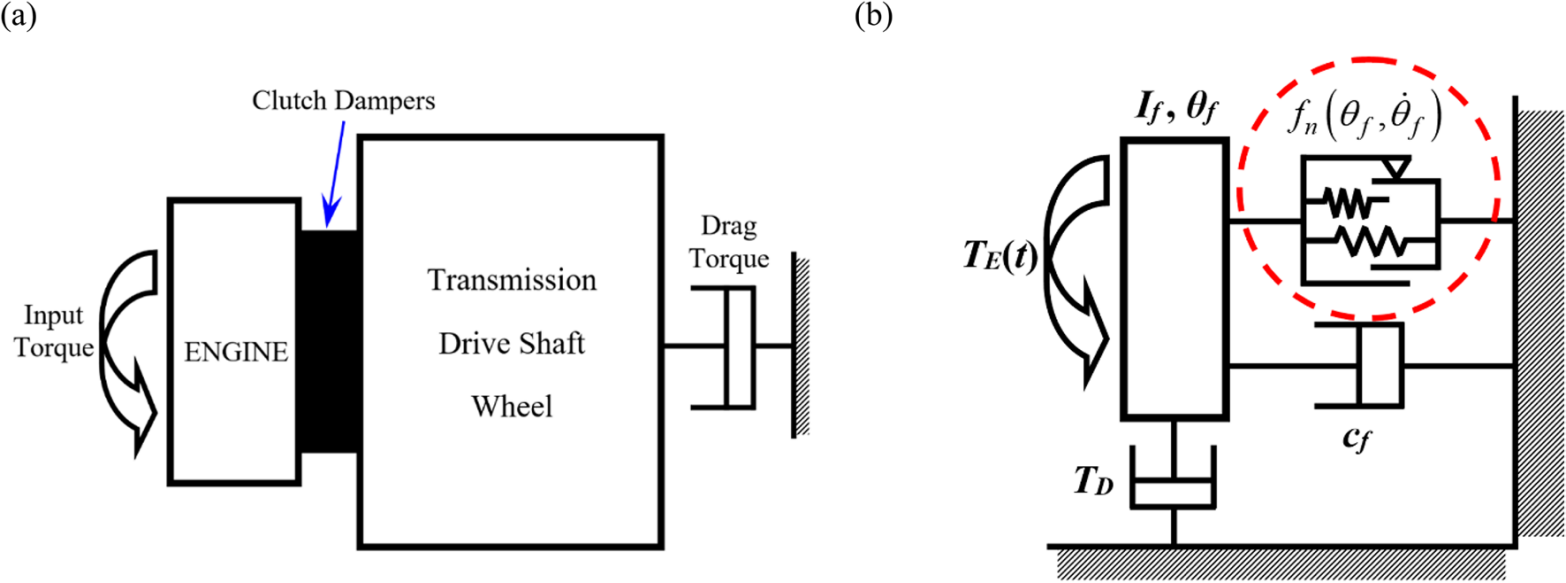
Practical system and its schematic diagram with piecewise type nonlinearities: (**a**) a simplified driveline system with multi-staged clutch dampers; (**b**) a torsional system with 1DOF affected by piecewise type nonlinearities.

Figure 1a illustrates the simple driveline system composed of the 1st mass for the engine and 2nd mass comprising the transmission, drive shaft, and wheel. Here, the 2nd mass is lumped with an equivalent inertia value derived from the inertia values of the gears, driveshafts, and wheels^6^. The input torque flows from the 1st mass into the 2nd mass, where the fluctuation of the input torque is isolated by the clutch dampers, as illustrated in Fig. 1a. By assuming that the inertia value of the 2nd mass is much greater than that of the 1st mass, the practical system can be considered as a single degree-of-freedom (DOF) system, as shown in Fig. 1b, where the 2nd mass is simply assumed as a ground. Thus, a nonlinear system with 1-DOF can be developed as a part of driveline focused on the rotational motion of the 1st mass and multi-staged clutch dampers based on prior studies^6,25^. Here, the 1st mass is considered as a lumped system, including the crankshaft and flywheel of the engine. To investigate the dynamic characteristics, the employed parameters for the torsional system shown in Fig. 1b are as follows: inertia of flywheel, *I*_*f*_ = 1.38 × 10^−1^ kg m^2^; viscous damping, *c*_*f*_ = 1.59 N m s/rad. In addition, the definitions of other parameters in Fig. 1b are input torque, *T*_*E*_, drag torque, *T*_*D*_, and clutch torque, ${f}_{n}\left({\theta }_{f},{\dot{\theta }}_{f}\right)$, which will be explained in detail later. Here, ${\theta }_{f}$ and ${\dot{\theta }}_{f}$ are the angular displacement and velocity of the flywheel (subscript *f*), respectively, as indicated in Fig. 1b.

### System modeling with piecewise type nonlinearities

**Figure 2:**
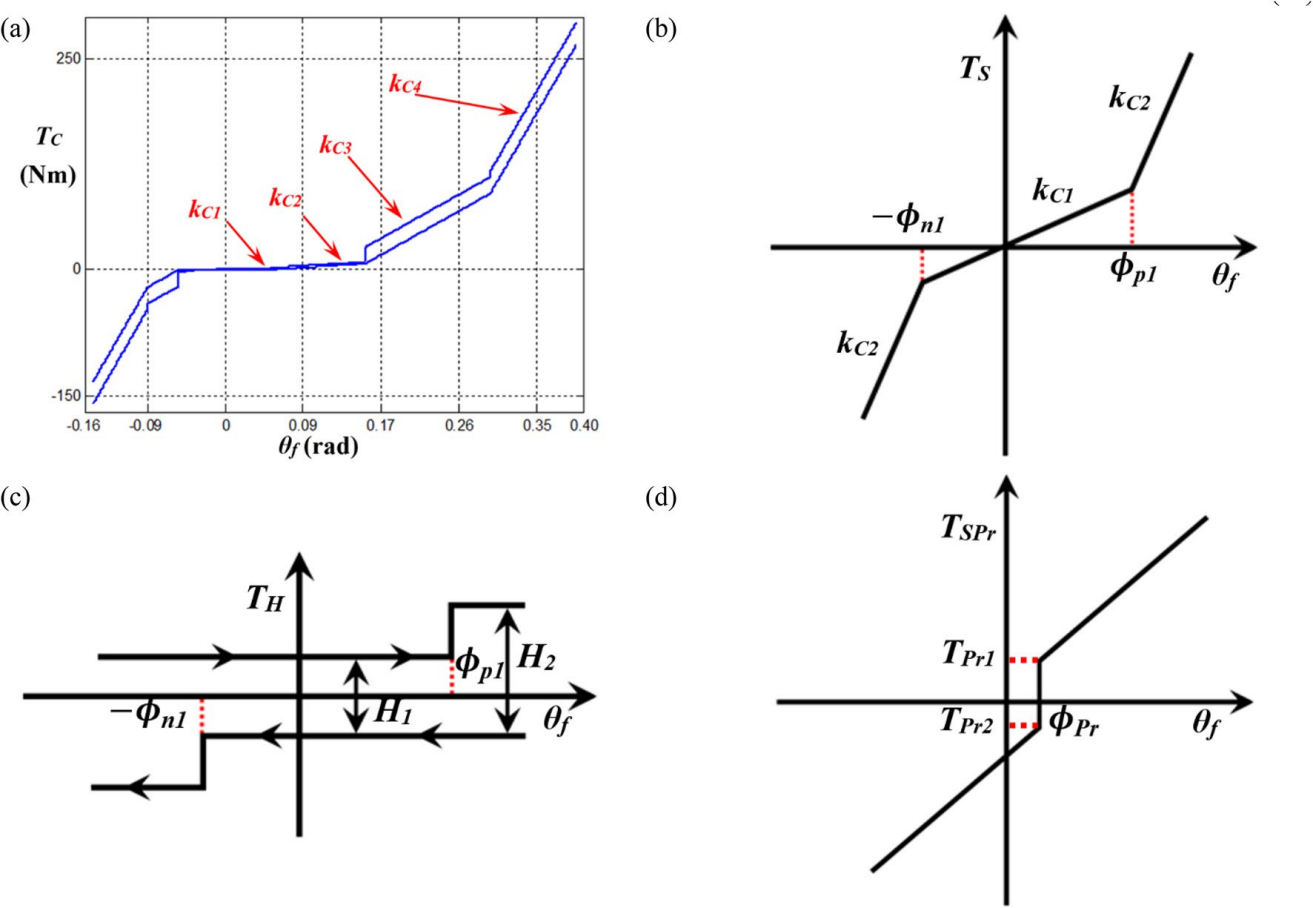
Asymmetric torque *T*_*C*_(*δ*_*1*_) profile induced by multi-staged clutch dampers: (**a**) the overall clutch torque profile; (**b**) the nonlinear force induced by multi-staged clutch springs; (**c**) the hysteresis induced by multi-staged friction force; (**d**) the preload based on asymmetric design concept.

The principle of piecewise-type nonlinearities, marked with a red dotted line in Fig. 1b, are depicted in Fig. 2. The basic profile of piecewise-type nonlinearities induced by the multi-staged clutch dampers is shown in Fig. 2a, where *k*_*Ci*_ (*i* = 1, 2, 3, 4) are the stages of stiffness. In general, the clutch force is affected by various factors, and the input torque is transferred from the engine to the rest of the driveline system^6^. First, the clutch torque *T*_*S*_ induced by multi-staged linear springs is considered, as shown in Fig. 2b, where ${\phi }_{p1}$ and $-\,{\phi }_{n1}$ are the transition angles on the positive and negative sides, respectively. Second, another clutch torque *T*_*H*_ is induced by the dry friction between the clutch plate and friction materials, as depicted in Fig. 2c. Third, the preload, *T*_*SPr*_, must also be considered based on the design concepts along with various practical conditions. As Fig. 2b–d show only the two stages of relevant components to clutch forces, more stages of clutch force components will be included for the practical system. The other parameters will be explained while deriving the mathematical description below.

For the employed piecewise-type nonlinearities, the nonlinear force ${f}_{n}\left({\theta }_{f},{\dot{\theta }}_{f}\right)$ (or *T*_*C*_) can be developed by mathematical formulation, as depicted in Fig. 2a^6,25^. First, the clutch torque ${T}_{S}\left({\theta }_{f}\right)$ is derived from the stiffness with a smoothing factor ${\sigma }_{C}$ employed as $1\times 1{0}^{3}$, as follows:

1 $${T}_{S}\left({\theta }_{f}\right)={k}_{C1}{\theta }_{f}+\frac{1}{2}\sum \limits_{i=2}^{N}\left({k}_{C\left(i\right)}-{k}_{C\left(i-1\right)}\right)\left({T}_{sp\left(i-1\right)}-{T}_{sn\left(i-1\right)}\right),$$

2 $${T}_{sp\left(i\right)}=\left({\theta }_{f}-{\phi }_{p\left(i\right)}\right)\left[\text{tan}h\left\{{\sigma }_{C}\left({\theta }_{f}-{\phi }_{p\left(i\right)}\right)\right\}+1\right],$$

3 $${T}_{sn\left(i\right)}=\left({\theta }_{f}+{\phi }_{n\left(i\right)}\right)\left[\text{tan}h\left\{{\sigma }_{C}\left({\theta }_{f}+{\phi }_{n\left(i\right)}\right)\right\}-1\right].$$

Here, *k*_*C*(*N*)_ (or *k*_*C*(*i*)_) is the *N*th (or *i*th) stage of the clutch stiffness (with subscript *N* or *i*), *T*_*sp*(*i*)_ (or *T*_*sn*(*i*)_) is the positive (or negative) side of the clutch torque induced by the stiffness at the *i*th stage (with subscript p or n), and ${\phi }_{p\left(i\right)}$ (or − ${\phi }_{n\left(i\right)}$) is the *i*th transition angle of the positive (or negative) side. Second, the clutch torque *T*_*H*_ induced by dry friction is derived by a smoothing factor ${\sigma }_{H}$ of 0.1.

4 $${T}_{H}\left({\theta }_{f},{\dot{\theta }}_{f}\right)=\frac{{H}_{\left(N\right)}}{2}\text{tan}h\left({\sigma }_{H}{\dot{\theta }}_{f}\right)+{\sum }_{i=2}^{N}\left(\frac{{H}_{\left(i\right)}}{4}-\frac{{H}_{\left(i-1\right)}}{4}\right)\left[{T}_{Hp\left(i-1\right)}+{T}_{Hn\left(i-1\right)}\right],$$

5 $${T}_{Hp\left(i\right)}=\text{tan}h\left\{{\sigma }_{C}\left({\theta }_{f}-{\phi }_{p\left(i\right)}\right)\right\}\left[1+\text{tan}h\left({\sigma }_{H}{\dot{\theta }}_{f}\right)\right],$$

6 $${T}_{Hn\left(i\right)}=\text{tan}h\left\{{\sigma }_{C}\left({\theta }_{f}+{\phi }_{n\left(i\right)}\right)\right\}\left[1-\text{tan}h\left({\sigma }_{H}{\dot{\theta }}_{f}\right)\right].$$

Here, *H*_*N*_ (or *H*_(*i*)_) is the *N*th (or *i*th) stage of hysteresis (with subscript *N* or *i*), and *T*_*Hp*(*i*)_ (or *T*_*Hn*(*i*)_) is the positive (or negative) side of the clutch torque induced by hysteresis at the *i*th stage (with subscript *p* or *n*). In addition to the torque calculated using Eqs. (1)–(6), the preload *T*_*Pr*_ must be considered as a function of ${\theta }_{1pr}$.

7 $${T}_{SPr}\left({\theta }_{1pr}\right)=\frac{1}{2}{T}_{Pr1}\left[\text{tan}h\left({\sigma }_{C}{\theta }_{1pr}\right)+1\right]+\frac{1}{2}{T}_{Pr2}\left[-\text{tan}h\left({\sigma }_{C}{\theta }_{1pr}\right)+1\right], {\theta }_{1pr}={\theta }_{f}-{\phi }_{Pr}.$$

Here, *T*_*SPr*_ is the total clutch torque induced by the pre-load; *T*_*Pr1*_ (or *T*_*Pr2*_) is the positive (or negative) torque induced by the pre-load; and ${\phi }_{Pr}$ is the angle located at the pre-load. Overall, the total clutch torque is estimated by the summation of ${T}_{S}\left({\theta }_{f}\right)$, ${T}_{H}\left({\theta }_{f},{\dot{\theta }}_{f}\right)$, and ${T}_{SPr}\left({\theta }_{1pr}\right)$ from Eqs. (1)–(7), as follows:

8 $${f}_{n}\left({\theta }_{f},{\dot{\theta }}_{f}\right)={T}_{C}\left({\theta }_{1pr},{\dot{\theta }}_{1pr}\right)={T}_{S}\left({\theta }_{1pr}\right)+{T}_{H}\left({\theta }_{1pr},{\dot{\theta }}_{1pr}\right)+{T}_{SPr}\left({\theta }_{1pr}\right).$$

The properties of the clutch torque ${f}_{n}\left({\theta }_{f},{\dot{\theta }}_{f}\right)$ (or *T*_*C*_) employed are listed in Table 1. Finally, the practical clutch torque profile can be calculated using Eqs. (1)–(8), as illustrated in Fig. 2a.

**Table 1:** Properties for the piecewise type nonlinearities based on the practical system.

Property	Stage	Value
Torsional stiffness, *k*_*Ci*_ (Linearized in a piecewise manner) (Nm/rad)	1	10.1
	2	61.8
	3	595.8
	4	1838.0
Hysteresis, *H*_*i*_ (Nm)	1	0.98
	2	1.96
	3	19.6
	4	26.5
Transition angle at positive side (*θ*_*f*_ > 0), $\phi_{pi}$ (rad)	1	0.05
	2	0.16
	3	0.30
	4	0.39
Transition angle at negative side (*θ*_*f*_ < 0), $\phi_{ni}$ (rad)	1	− 0.04
	2	− 0.05
	3	− 0.09
	4	− 0.15

### Basic equation and development of HBM

The basic equation of motion for the torsional system, which includes the nonlinear function ${f}_{n}\left({\theta }_{f},{\dot{\theta }}_{f}\right)$ shown in Fig. 1b, is derived as follows:

9 $${I}_{f}{\ddot{\theta }}_{f}\left(t\right)+c{\dot{\theta }}_{f}\left(t\right)+{f}_{n}\left({\theta }_{f},{\dot{\theta }}_{f}\right)={T}_{E}\left(t\right)-{T}_{D}.$$

Here, *T*_*E*_(*t*) and *T*_*D*_ are the sinusoidal input and drag torques, respectively. In this study, the input torque is given by Fourier coefficients based on the measured data as follows:

10 $${T}_{E}\left(t\right)={T}_{m}+\sum \limits_{i=1}^{{N}_{\mathit{max} }}{T}_{pi}\text{cos}\left(i{\omega }_{p}t+{\varphi }_{pi}\right).$$

Here, *T*_*m*_ and *T*_*pi*_ are the mean and alternating parts of the input torque, respectively; ${\omega }_{p}$ and ${\varphi }_{pi}$ are the excitation frequency and phase angle, respectively; and *N*_max_ is the maximum number of harmonics correlated with the harmonic index of the HBM. The input torque profiles employed are listed in Table 2.

**Table 2:** Employed input torque profiles from the engine dynamometer test.

Torque component		
Magnitude (Nm)		Phase (rad)
*T*_*M*_	*T*_*p1*_	
168.9	251.5	− 1.93

In this study, the drag torque is assumed as *T*_*D*_ = *T*_*m*_ under steady-state conditions. To find out the nonlinear dynamic behaviors, this study will employ the harmonic balance method (HBM) since the HBM generally give the frequency and time domain solutions simultaneously under the steady-state and frequency sweeping conditions. Also, while the simulation is conducted, stability conditions can be efficiently determined by employing the Hill’s method^5,6,27^. The Galerkin scheme of Eq. (10) is expressed as follows:

11 $$- \omega^{2} m\underline{\underline{{{\mathbf{HP}}}}}^{{\prime\prime}} \underline{{\theta_{c} }} + \omega c\underline{\underline{{{\mathbf{HP}}}}}^{{\prime}} \underline{{\theta_{c} }} + \underline{{{\mathbf{f}}_{{\mathbf{n}}} }} \left( {\underline{{{{\varvec{\uptheta}}}_{{\mathbf{f}}} }} ,\underline{{{\dot{\mathbf{\theta }}}_{{\mathbf{f}}} }} } \right) - \underline{{{\mathbf{F}}_{{\mathbf{E}}} }} \left( t \right) = \underline{0} .$$

Then, its corresponding formulae are defined as follows:

12 $$\underline{{{{\varvec{\uptheta}}}_{{\mathbf{f}}} }} \left( t \right) = \underline{\underline{{\mathbf{H}}}} \underline{{{{\varvec{\uptheta}}}_{{\mathbf{c}}} }} ,\;\underline{{{{\varvec{\uptheta}}}_{{\mathbf{f}}} }} \left( t \right) = \left[ {\theta_{f} \left( {t_{0} } \right)\quad \theta_{f} \left( {t_{1} } \right) \ldots \theta_{f} \left( {t_{m - 2} } \right)\quad \theta_{f} \left( {t_{m - 1} } \right)} \right]^{T} ,$$

13 $$\underline{{{{\varvec{\uptheta}}}_{{\mathbf{c}}} }} = \left[ {\begin{array}{*{20}c} {\theta_{m} } & {\theta_{a\left( 1 \right)} } & {\theta_{b\left( 1 \right)} } & \cdots & {\theta_{a\left( k \right)} } & {\theta_{b\left( k \right)} } & \cdots & {\theta_{{a\left( {\eta N_{{\max { }}} } \right)}} } & {\theta_{{b\left( {\eta N_{{\max { }}} } \right)}} } \\ \end{array} } \right]^{{\text{T}}} ,$$

14 $$\underline{{\mathbf{H}}} = \left[ {\begin{array}{*{20}c} 1 & \cdots & {{\text{cos}}\left( {k\psi_{0} } \right)} & {{\text{sin}}\left( {k\psi_{0} } \right)} & \cdots \\ 1 & \cdots & {{\text{cos}}\left( {k\psi_{1} } \right)} & {{\text{sin}}\left( {k\psi_{1} } \right)} & \cdots \\ { } & \ddots & { } & { } & \ddots \\ 1 & \cdots & {{\text{cos}}\left( {k\psi_{N - 2} } \right)} & {{\text{sin}}\left( {k\psi_{N - 2} } \right)} & \cdots \\ 1 & \cdots & {{\text{cos}}\left( {k\psi_{N - 1} } \right)} & {{\text{sin}}\left( {k\psi_{N - 1} } \right)} & \cdots \\ \end{array} } \right],\; \underline{\underline{{\mathbf{H}}}}^{\prime } = \omega \underline{\underline{{\mathbf{H}}}} \underline{\underline{{\mathbf{P}}}}^{\prime } , \; \underline{\underline{{\mathbf{H}}}}^{\prime } = - \omega^{2} \underline{\underline{{\mathbf{H}}}} \underline{\underline{{\mathbf{P}}}}^{\prime } ,$$

15 $$\underline{{{\mathbf{\underline {P} }}}}^{{\prime}} = \left[ {\begin{array}{*{20}c} 0 & & & \\ & \ddots & & \\ & & {\left[ {\begin{array}{*{20}c} 0 & k \\ { - k} & 0 \\ \end{array} } \right]} & \\ & & & \ddots \\ \end{array} } \right], \;\underline{{{\mathbf{\underline {P} }}}}^{{^{\prime\prime}}} = \left[ {\begin{array}{*{20}c} 0 & & & \\ & \ddots & & \\ & & {\left[ {\begin{array}{*{20}c} {k^{2} } & 0 \\ 0 & {k^{2} } \\ \end{array} } \right]} & \\ & & & \ddots \\ \end{array} } \right].$$

Likewise, its nonlinear and input functions are defined as follows:

16 $$\underline{{{\mathbf{f}}_{{\mathbf{n}}} }} \left( {\underline{{{{\varvec{\uptheta}}}_{{\mathbf{f}}} }} ,\underline{{{\dot{\mathbf{\theta }}}_{{\mathbf{f}}} }} } \right) = \underline{\underline{{\mathbf{H}}}} \underline{{{\mathbf{f}}_{{{\mathbf{nc}}}} }} , \underline{{{\mathbf{F}}_{{\mathbf{E}}} }} \left( t \right) = \underline{\underline{{\mathbf{H}}}} \underline{{{\mathbf{F}}_{{{\mathbf{Ec}}}} }} ,$$

17 $$\underline{{{\mathbf{f}}_{{{\mathbf{nc}}}} }} = \left[ {\begin{array}{*{20}c} {f_{m} } & {f_{a\left( 1 \right)} } & {f_{b\left( 1 \right)} } & \cdots & {f_{a\left( k \right)} } & {f_{b\left( k \right)} } & \cdots & {f_{{a\left( {\eta N_{\max } } \right)}} } & {f_{{b\left( {\eta N_{\max } } \right)}} } \\ \end{array} } \right]^{{\text{T}}} ,$$

18 $$\underline{{{\mathbf{F}}_{{{\mathbf{Ec}}}} }} = \left[ {\begin{array}{*{20}c} {F_{m} } & {F_{a\left( 1 \right)} } & {F_{b\left( 1 \right)} } & \cdots & {F_{a\left( k \right)} } & {F_{b\left( k \right)} } & \cdots & {F_{{a\left( {\eta N_{\max } } \right)}} } & {F_{{b\left( {\eta N_{\max } } \right)}} } \\ \end{array} } \right]^{{\text{T}}} .$$

The relevant variables used are $\overline{\omega } t = \psi$ and $\overline{\omega } = \frac{\omega }{{\omega_{n} }}$, where ${\omega }_{n}$ is the non-dimensionalized time scale and normalized frequency value with the natural frequency; $T=\eta \tau$ is the concerned time period with respect to $0\le t<T$ → $0\le \psi <\frac{2\pi }{{\omega }_{n}}$; $\eta$ is the sub-harmonic index; $\tau$ is the fundamental excitation frequency; and *k* is the incremental index as $k = \omega_{n} , 2\omega_{n} , 3\omega_{n} \ldots$. By employing the relationship between $\dot{\theta }\left( t \right) = \frac{d\theta }{{dt}} = \overline{\omega } \frac{d\theta }{{d\psi }} = \overline{\omega } \theta^{\prime}$ and $\ddot{\theta }\left( t \right) = \overline{\omega }^{2} \theta^{\prime\prime}$, the overall Galerkin scheme of the basic equation of Eq. (11) is expressed as follows:

19 $${\underline{\underline{{\mathbf{H}}}}} {\underline{\underline{{{\varvec{\Psi}}}}}} = \underline {0} , {\mathbf{\underline {\Psi } }} = - \overline{\omega }^{2} m\underline{\underline{{\mathbf{P}}}}^{\prime } \underline{{{{\varvec{\uptheta}}}_{{\mathbf{c}}} }} + \overline{\omega } c\underline{\underline{{\mathbf{P}}}}^{\prime } \underline{{{{\varvec{\uptheta}}}_{{\mathbf{c}}} }} + \underline{{{\mathbf{f}}_{{{\mathbf{nc}}}} }} - \underline{{{\mathbf{F}}_{{{\mathbf{Ec}}}} }} = \underline {0} .$$

To determine the solutions for ${\underline{{{\varvec{\uptheta}}}_{\mathbf{c}}}}$ and $\varpi$ for each step, the Newton–Raphson method is implemented under the condition ${\underline{{\varvec{\Psi}}}}\to \underline{0}$, where ${\underline{{\varvec{\Psi}}}}$ is considered as a function of ${\underline{{{\varvec{\uptheta}}}_{\mathbf{c}}}}$ and $\varpi$ such that ${\underline{\varvec{\Psi}}}\left({\underline{\varvec{\uptheta}}_{\mathbf{c}}},\varpi \right)$. A detailed explanation about these parameters and their use can be found in previous studies^6^.

## Initial results with frequency upsweeping condition

**Figure 3:**
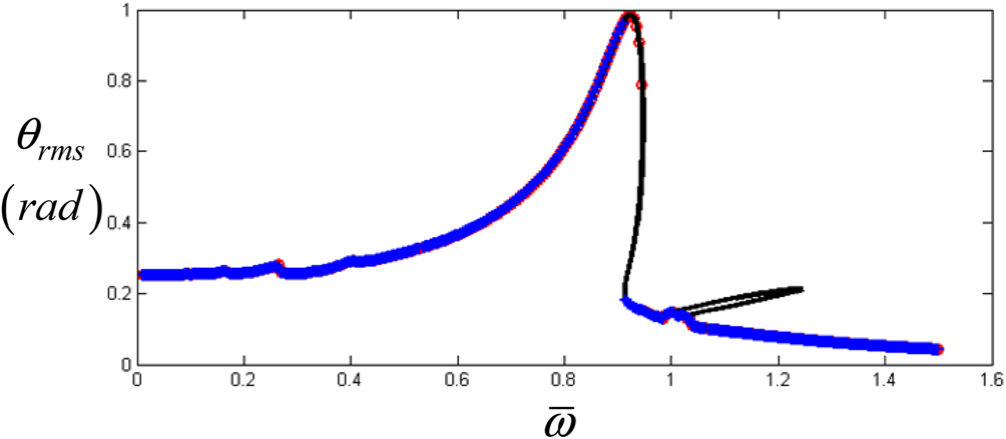
Initial results of nonlinear frequency responses with RMS by comparing the HBM and NS. Key: black line, the HBM result with *ζ* = 0.05; red open circle, NS under the frequency upsweeping; blue plus, NS under the frequency down-sweeping.

**Figure 4:**
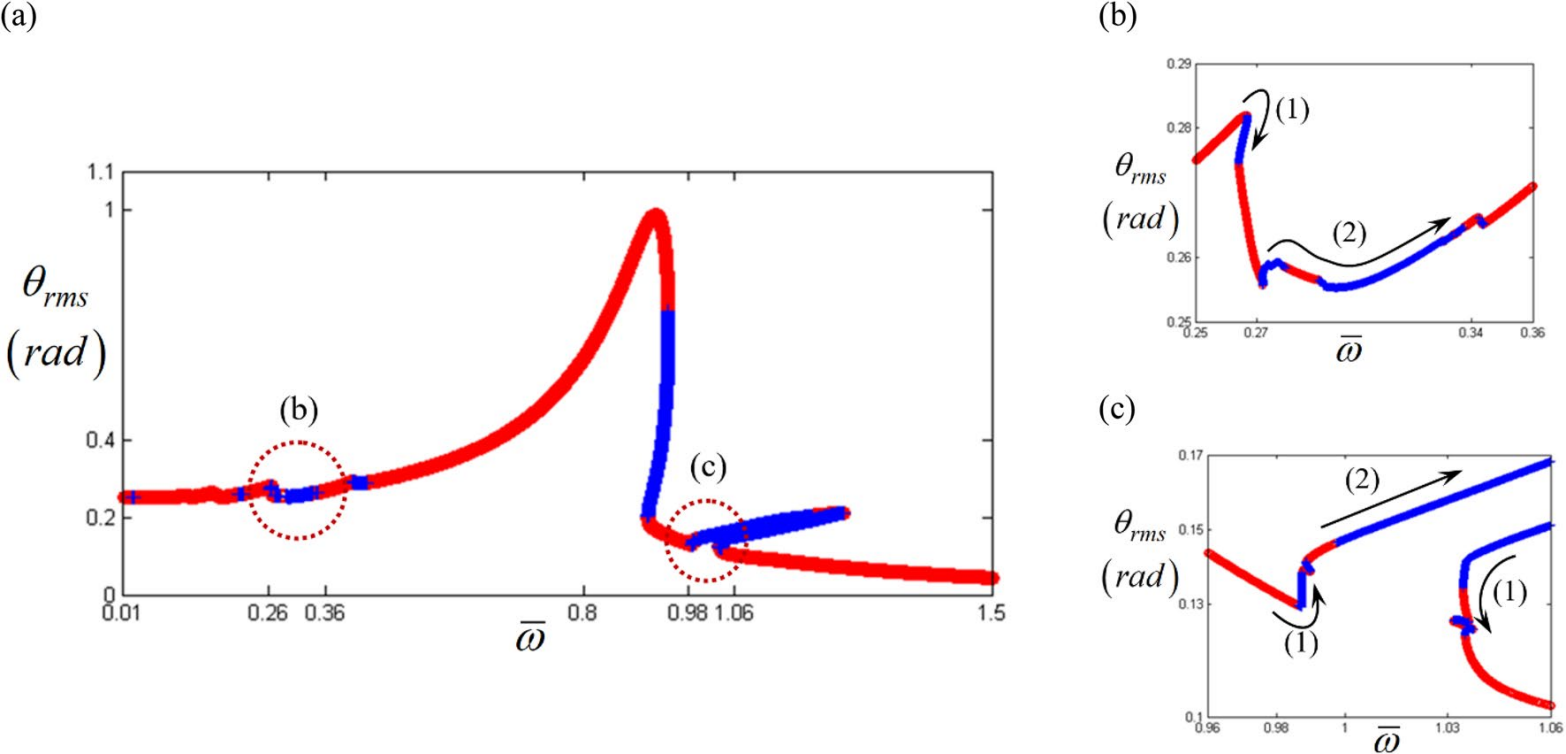
Nonlinear frequency response with RMS reflecting the stability conditions under the frequency upsweeping: (**a**) RMS values with super- and sub-harmonic regimes; (**b**) super-harmonic response area; (**c**) sub-harmonic response area. Key: red open circle, stable solutions; blue plus, unstable solutions.

Figures 3 and 4 show the initial results obtained by the HBM with $\eta$ = 2 and *N*_max_ = 24 under the frequency upsweeping condition. Figure 3 compares the results based on two different methods such as the HBM and the numerical simulation (NS), which reflects good correlations except for the sub-harmonic resonance area. Moreover, the Hill’s method is employed to determine the stability condition as shown in Fig. 4^5,6,17^. For this study, the valid components of the input torque vector with $\eta$ = 2 and *N*_max_ = 24 are given as ${F}_{m}=168.9$, ${F}_{a\left(2\right)}=- 87.97$, and ${F}_{b\left(2\right)}=235.65$ in Eq. (18). Here, the employed values, $\eta$ =2 and *N*_max_ = 24 were enough to project the nonlinear effect when the simulations from the HBM are compared with the numerical simulation (NS)^5,6,27^. In general, the employment of values for $\eta$ and *N*_max_ differs depending on the various types of nonlinearities. Also, the other issues such as a convergence and number of index numbers for $\eta$ and *N*_max_ can be referred^5,6,27^.

To examine the sub-harmonic responses, the components of the input torque pertaining to the sub-harmonic locations, such as ${F}_{a\left(1\right)}$ and ${F}_{b\left(1\right)}$, are ${F}_{a\left(1\right)}=\varepsilon {F}_{a\left(2\right)}$ and ${F}_{b\left(1\right)}=\varepsilon {F}_{b\left(2\right)}$. Here, $\varepsilon$ is taken as equal to $1\times 1{0}^{-5}$. To obtain the sub-harmonic responses, a small range of excitation input values are artificially employed^18^. Figure 4a shows the simulated results, including both super- and sub-harmonic resonances, marked with red dotted circles. In addition, the resonant areas of (b) and (c) in the red dotted circles are more concentrated in Fig. 4b,c, respectively. When the super- and sub-harmonic resonances are examined, the stability variational conditions, such as stable to unstable (STU) and unstable to stable (UTS) conditions, are clearly observed. In addition, the STU and UTS conditions are simultaneously considered with the direction of the arc-length solution, as indicated by (1) and (2) in Fig. 4b,c. For example, the regimes indicated as (1) in Fig. 4b,c show that the directions of solutions are changed from facing the same direction as frequency upsweeping to facing the opposite direction under STU or UTS conditions.

However, the regimes indicated as (2) always follow the same direction as frequency upsweeping for both STU and UTS conditions. Based on prior studies, the regimes marked as (1) are expected to clearly show that the jumping phenomenon occurs; the phenomenon is normally known as the saddle-node points that are observed at slightly different locations based on the frequency upsweeping or down-sweeping conditions. In general, a representative saddle-node bifurcation is clearly observed in the primary resonant area, as shown in Fig. 4a. However, regarding the regimes (2) with unstable conditions, as shown in Fig. 4b,c, it is difficult to anticipate specifically whether the simulated results under STU occur in the practical system or they just project theories from the HBM using Hill’s method. Thus, more techniques must be considered to understand the nonlinear dynamic behaviors with respect to the bifurcation characteristics.

## Examination of primary, super-, and sub-harmonic resonances

**Figure 5:**
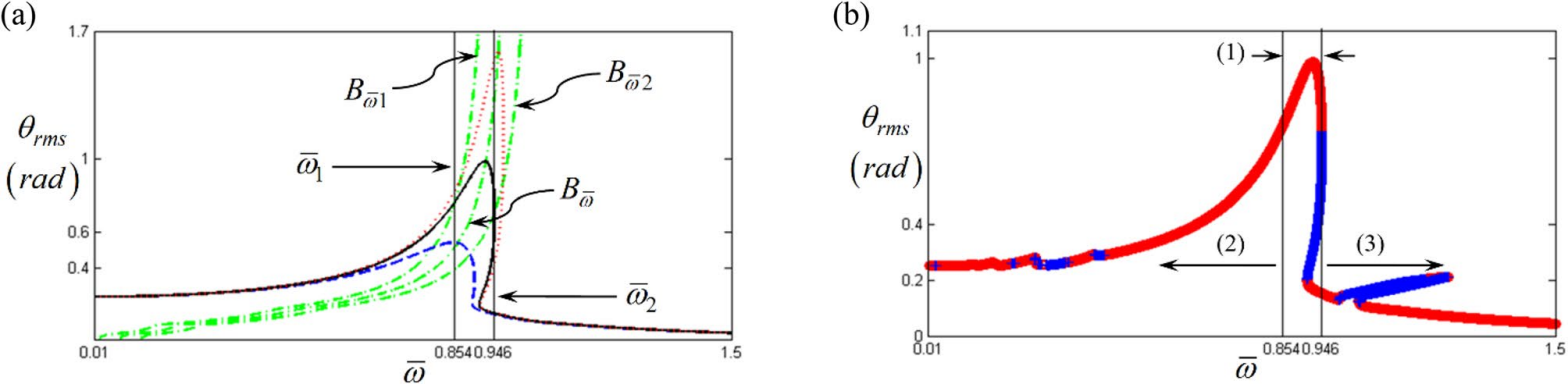
Nonlinear frequency responses focused on super-harmonic regimes with RMS values. Key: red open circle, stable solutions; blue plus, unstable solutions.

As a first step to examine the nonlinear dynamic behaviors, different resonant areas such as primary, super-, and sub-harmonic resonances are determined, as shown in Fig. 5. To define the various resonant regimes, basic linear concepts for the half-power method can be employed^26^. For example, the relationships between the Q factor location and the half power location lead to the following basic equations:

20 $$\frac{1}{2\zeta } = \frac{{\omega_{n} }}{\Delta \omega },\;\Delta \omega = \omega_{2} - \omega_{1} ,$$

21 $$\zeta = \frac{{\Delta \overline{\omega }_{n} }}{2},\;\Delta \overline{\omega }_{n} = \frac{{\Delta \omega_{n} }}{{\omega_{n} }},$$

22 $$\overline{\omega }_{1} \approx \overline{\omega }_{n} - \frac{{\Delta \overline{\omega }_{n} }}{2} = \overline{\omega }_{n} - \zeta ,$$

23 $$\overline{\omega }_{2} \approx \overline{\omega }_{n} + \frac{{\Delta \overline{\omega }_{n} }}{2} = \overline{\omega }_{n} + \zeta .$$

Here, ${\omega }_{n}$, ${\omega }_{1}$, and ${\omega }_{2}$ are the natural frequency and the lower and higher values of half power locations for the linear system, respectively. In addition, $\overline{\omega }_{i}$ (with *i* = 1, 2) is the normalized frequency value of ${\omega }_{n}$. Thus, this study employs the same concepts to define different resonant areas based on Eqs. (22) and (23). Figure 5a illustrates the comparisons of the HBM results with various damping ratios using backbone curves. Here, $B_{{\overline{\omega } }}$ is the basic backbone curve, which is estimated under no input or damping conditions^27^. In addition to the basic $B_{{\overline{\omega } }}$ curve, two more backbone curves transferred from $B_{{\overline{\omega } }}$ along the $\overline{\omega }$ axis can be calculated as follows:

24 $$B_{{\overline{\omega } 1}} = B_{{\overline{\omega } }} - \zeta , B_{{\overline{\omega } 2}} = B_{{\overline{\omega } }} + \zeta .$$

Figure 5a shows three cases of HBM results for $\zeta$ = 0.03, 0.05, and 0.1, via a comparison of the backbone curve $B_{{\overline{\omega } }}$ and the transferred backbone curves. Here, assuming that the concepts in Eq. (20)–(23) for the linear system can be approximately employed for the nonlinear system, the cross points of $B_{{\overline{\omega } 1}}$ and $B_{{\overline{\omega } 2}}$ with respect to the HBM result with $\zeta$ = 0.05 are considered as $\overline{\omega }_{1}$ and $\overline{\omega }_{2}$ as indicated in Fig. 5a,b. Based on Eq. (24), the local resonances are obtained, which are normally defined as primary, super-, and sub-harmonic resonances, along with different frequency response regimes. Thus, when the bifurcation point (BP) is estimated by focusing on the frequency upsweeping condition in this study, the simulation can simultaneously recognize their relevant regimes for the primary, super-, and sub-harmonic responses by employing the values $\overline{\omega }_{1}$ and $\overline{\omega }_{2}$ calculated from Eq. (24). Figure 5b shows the different resonant areas referenced by $\overline{\omega }_{1}$ and $\overline{\omega }_{2}$. For example, the areas marked with (1), (2), and (3) indicate the primary, super, and sub-harmonic resonances, respectively. In addition, the primary resonance is normally affected by the saddle-node BP, as shown in Fig. 5b.

## Investigation of saddle-node bifurcation point

**Figure 6:**
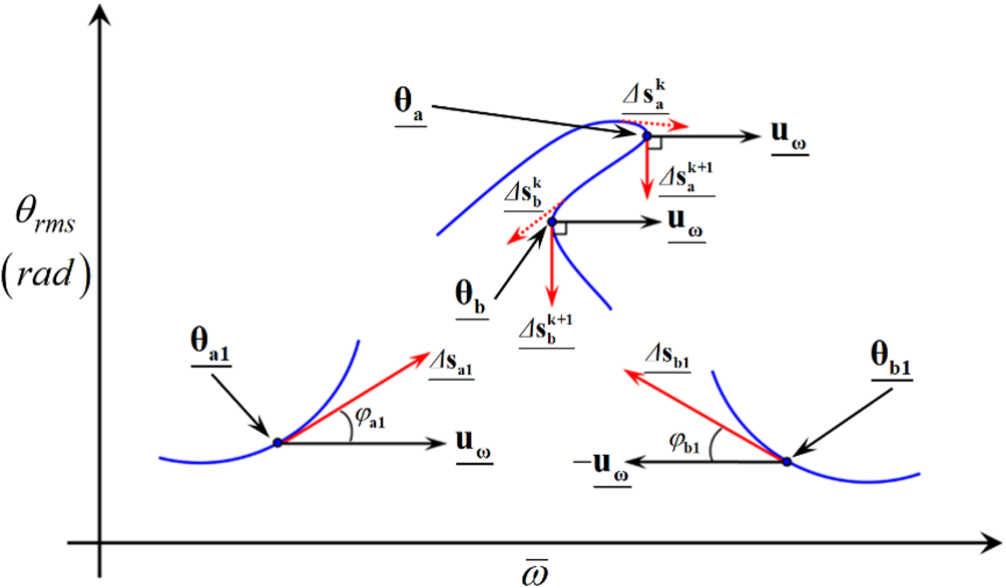
Particular points of nonlinear frequency responses by considering the angles between the arc-length and unit vectors.

As a second step to determine the BPs, the locations where the stability conditions vary can be determined. In general, bifurcation occurs when the real parts of the eigenvalues are zero^28^. In addition, if the variational condition of arc-length directions at a specific point is examined, then the bifurcation characteristics in the next steps of the solutions can be determined in terms of saddle-node or other types of bifurcations. To determine the saddle-node BP, the direction of the arc-length vector can be estimated. Here, by assuming the physical dynamic behaviors at the saddle-node BP are directly correlated with the jumping phenomena, as clearly observed in Figs. 4 and 5, the angle at this point is deduced to be 90° under STU or UTS conditions. To calculate and determine the saddle-node point, the angle between the arc-length and unit vectors can be estimated. Figure 6 depicts the various cases of arc-length vectors and their angles with unit vectors.

Here, the unit vectors with the $2\eta {N}_{\mathit{max} }+1$ number of components along with the size of HBM matrices and vectors are defined as follows:

25 $$\underline{{\mathbf{u}}_{\omega }} ={\left[\begin{array}{ccc}0& \cdots & 1\end{array}\right]}^{T}, -\underline{{\mathbf{u}}_{\omega }}={\left[\begin{array}{ccc}0& \cdots & -1\end{array}\right]}^{T}.$$

Thus, the unit vectors are always in the horizontal direction, which are referenced to calculate the angles of the arc-length vector $\underline{{\Delta s_{i} }}$ (with *i* = a1, b1, and a) from ${\underline{{\mathbf{u}}_{\omega }}}$ or $-{\underline{{\mathbf{u}}_{\omega }}}$ at each step, as indicated in Fig. 6. Here, ${\underline{{{\varvec{\uptheta}}}_{a1}}}$, ${\underline{{{\varvec{\uptheta}}}_{b1}}}$, ${\underline{{{\varvec{\uptheta}}}_{a}}}$, and ${\underline{{{\varvec{\uptheta}}}_{b}}}$ are certain points of solution that determine the BPs. To calculate the angles constructed by each arc-length with the unit vectors, the basic concept of inner product can be used, as follows:

26 $${\varphi }_{i}=\frac{\langle \underline{{\mathbf{u}}_{\omega }},\,{\underline{\Delta {\mathbf{s}}_{i}}\rangle }}{\left|\underline{{{\mathbf{u}}_{\omega }}}\right|\left|{\underline{\Delta {\mathbf{s}}_{i}}}\right|}, i =\text{ a}1,\text{ b}1,\text{ a and b}.$$

While ${\varphi }_{i}$ is estimated, $\underline{{\mathbf{u}}_{\omega }}$ or $-\underline{{\mathbf{u}}_{\omega }}$ must be chosen based on the frequency up- or down-sweeping conditions, respectively. For the locations of $\underline{{{{\varvec{\uptheta}}}_{a1}}}$ and $\underline{{{{\varvec{\uptheta}}}_{b1}}}$, the composed angles ${\varphi }_{a1}$ (or ${\varphi }_{b1}$) between ${\underline{\Delta {\mathbf{s}}_{a1} }}$ (or ${\underline{\Delta {\mathbf{s}}_{b1} }}$) and ${\underline{{\mathbf{u}}_{\omega } }}$ (or $- {\underline{{\mathbf{u}}_{\omega } }}$) are much less than 90°. However, other points, such as ${\underline{{{{\varvec{\uptheta}}}_{a} }}}$ and ${\underline{{{{\varvec{\uptheta}}}_{b} }}}$ show much stiffer variational changes in angles, then their angles approach 90°. For example, when the arc-length vector around ${\underline{{{{\varvec{\uptheta}}}_{a} }}}$ changes its direction from the frequency upsweeping to the opposite direction, ${\underline{{\Delta {\mathbf{s}}_{a}^{k} }}}$ at the prior step finally reaches $\underline{{\Delta {\mathbf{s}}_{a}^{k + 1} }}$ at the current step, where the composed angle between $\underline{{\Delta {\mathbf{s}}_{a}^{k + 1} }}$ and $\underline{{{\mathbf{u}}_{\omega } }}$ becomes 90°. Again, when the arc-length vector around $\underline{{{{\varvec{\uptheta}}}_{b} }}$ changes its direction from the opposite to the frequency upsweeping direction, $\underline{{\Delta {\mathbf{s}}_{b}^{k} }}$ goes to $\underline{{\Delta {\mathbf{s}}_{b}^{k + 1} }}$, which is at 90° with $\underline{{{\mathbf{u}}_{\omega } }}$. Based on the given variational conditions, the angle index can be determined as follows:

27 $$\Delta \varphi = \left| {\frac{\pi }{2} - \left| {\varphi_{i} } \right|} \right| \;\left( {{\text{with }}i{ } = {\text{ a}}1,{\text{ b}}1,{\text{ a}},{\text{ and b}}} \right).$$

Because ${\varphi }_{i}$ is calculated numerically while the HBM is conducted, the angles obtained are approximately 90°. Thus, the absolute value $\left|{\varphi }_{i}\right|$ is used to determine the difference from 90°. Here, $\left|{\varphi }_{i}\right|$ is considered as approximately 90° if $\Delta \varphi < \varepsilon_{\varphi }$, where the index number $\varepsilon_{\varphi }$ is π/180 rad. Thus, if the condition $\Delta \varphi < \varepsilon_{\varphi }$ is satisfied, this point is determined as a saddle-node point. Overall, the examination of $\Delta \varphi$ with respect to the stability conditions—namely, STU and UTS—leads to two different bifurcation conditions, such as saddle-node and other types of bifurcations, as summarized in Table 3.

**Table 3:** Bifurcation characteristics along with various angles between the arc-length and unit vectors under the stability variational conditions.

Stability conditions	Conditions of $\Delta \varphi$	Characteristics of bifurcation points
Stable → unstable (STU)	$\Delta \varphi < \varepsilon_{\varphi }$	Saddle-node
	$\Delta \varphi > \varepsilon_{\varphi }$	Period-doubling cascade/quasi-periodic/chaotic
Unstable → stable (UTS)	$\Delta \varphi < \varepsilon_{\varphi }$	Saddle-node
	$\Delta \varphi > \varepsilon_{\varphi }$	Stable

**Figure 7:**
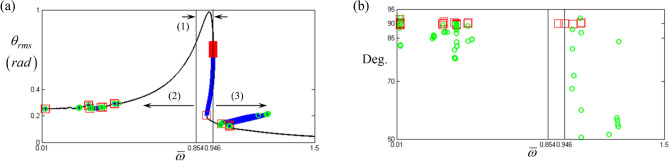
Comparisons of bifurcation points with stability conditions: (**a**) various bifurcation points along with primary, super-, and sub-harmonic resonant areas; (**b**) relationship of bifurcation points to the angles between the arc-length and unit vectors. Key: red open square, saddle-node points; green open circle, bifurcation points other than saddle-node; black line, HBM solutions.

Figure 7a clearly shows the saddle-node and other bifurcation points determined by Eqs. (25)–(27). In addition, the distribution of $\Delta \varphi$ under the frequency upsweeping condition is shown in Fig. 7b, where the saddle-node points indicated by red rectangles are located near 90°. Except for the saddle-node points, other BPs illustrated with green circles are located below or away from 90°; their relationships are described in Eq. (27).

**Figure 8:**
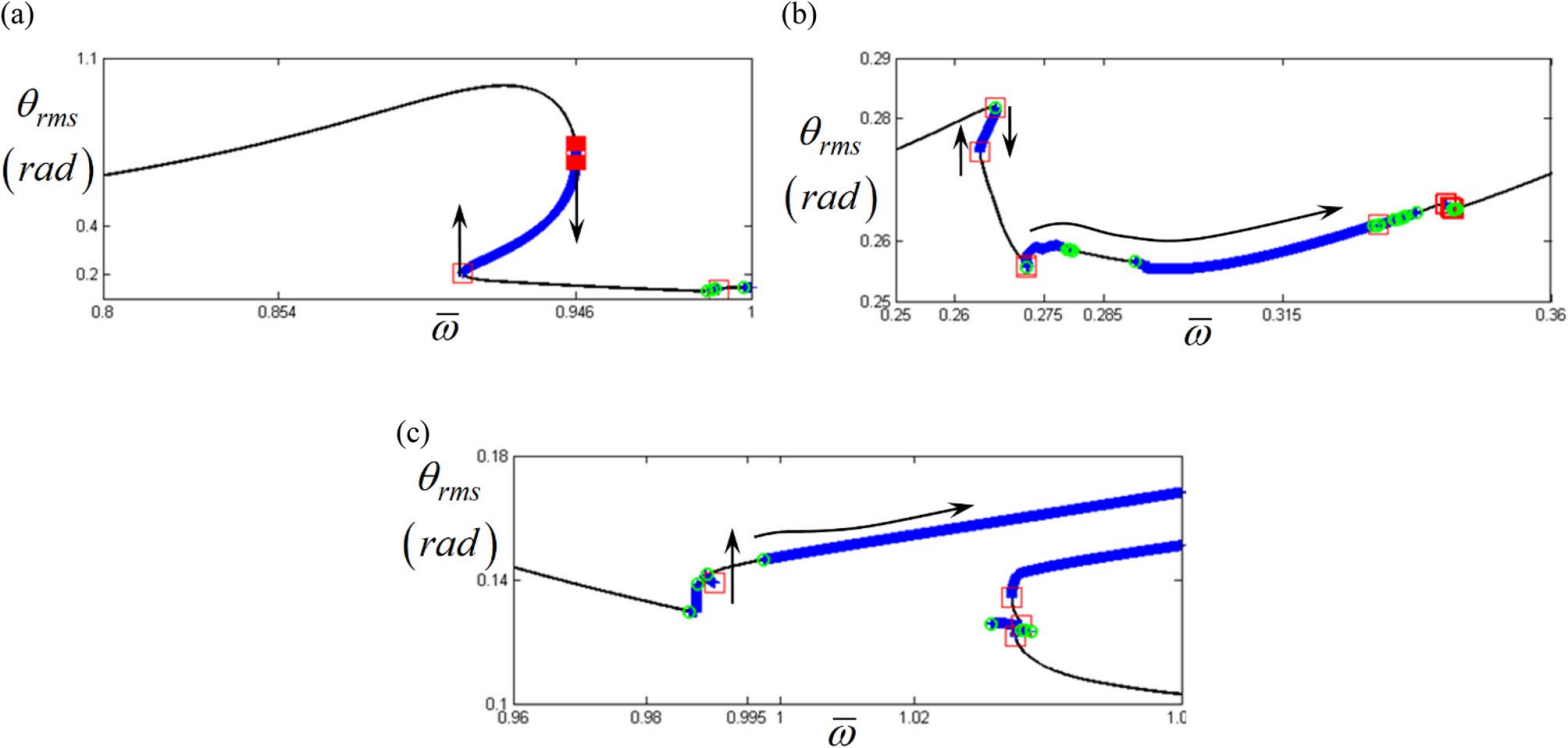
Bifurcation points along with various resonant areas: (**a**) bifurcation points at the primary resonance; (**b**) bifurcation points at the super-harmonic regime; (**c**) bifurcation points at the sub-harmonic regime. Key: red open square, saddle-node point; green open circle, bifurcation points other than saddle-node; black line, stable solutions; blue plus, unstable solutions.

Figure 8a–c show the BPs in more detail by focusing on the primary, super-, and sub-harmonic resonant areas, as indicated by (1), (2), and (3) in Fig. 7a, respectively. From the results illustrated in Fig. 8, it can be observed that the saddle-node BPs are located at the jump phenomena points, whereas the other BPs follow the frequency upsweeping direction. In addition, the multiple saddle-node points found at the primary resonance are due to numerical problems during the HBM process, which will be further investigated in a future work.

## Examination of eigenvalues for the bifurcation characteristics

**Figure 9:**
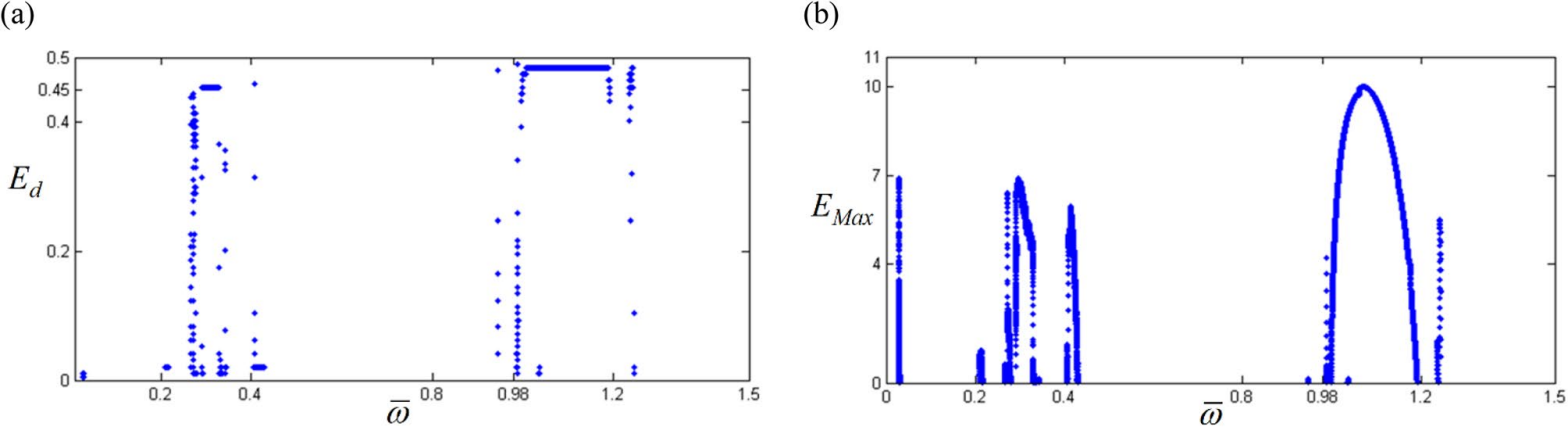
Positive values in the real part of eigenvalues: (**a**) ${E}_{d}$ in the real part of eigenvalues; (**b**) ${E}_{Max}$ in the real part of eigenvalues.

The saddle-node BP is clearly revealed along with the estimation of the arc-length direction based on frequency upsweeping in this study, as shown in Figs. 5, 7, and 8. However, other BPs relevant to the period-doubling, quasi-periodic, and chaotic cascades are not clearly observed from the previous techniques. To analyze other types of bifurcations, the real parts of eigenvalues Re(*λ*) will be examined. To determine the relationships between Re(*λ*) and the bifurcation characteristics, the distribution of positive Re(*λ*) with respect to the overall number of Re(*λ*) is first examined. The positive Re(*λ*) is correlated with the unstable condition of the system responses. Second, a reference value of Re(*λ*), such as the maximum positive Re(*λ*), is investigated over the range of frequencies for the super- and sub-harmonic resonances. Figure 9 shows the two properties previously described. Figure 9a shows the distribution of positive Re(*λ*) values over all numbers of Re(*λ*), calculated as follows:

28 $$E_{d} = \frac{{Numbers\; of\; positive \;{\text{Re}} \left( {\lambda_{i} } \right)}}{{Total \;numbers\; of\; {\text{Re}} \left( {\lambda_{j} } \right)}}\; with \;i, j = 1, 2, \ldots .$$

In addition, Fig. 9b shows the positive maximum values of Re(*λ*) over the range of our simulation. Here, *E*_*max*_ is defined as the maximum positive Re(*λ*). The results in Fig. 9a,b show that significant values around the super- and sub-harmonic resonances are distributed, especially when they are compared with the HBM results given in Figs. 5, 7, and 8.

**Figure 10:**
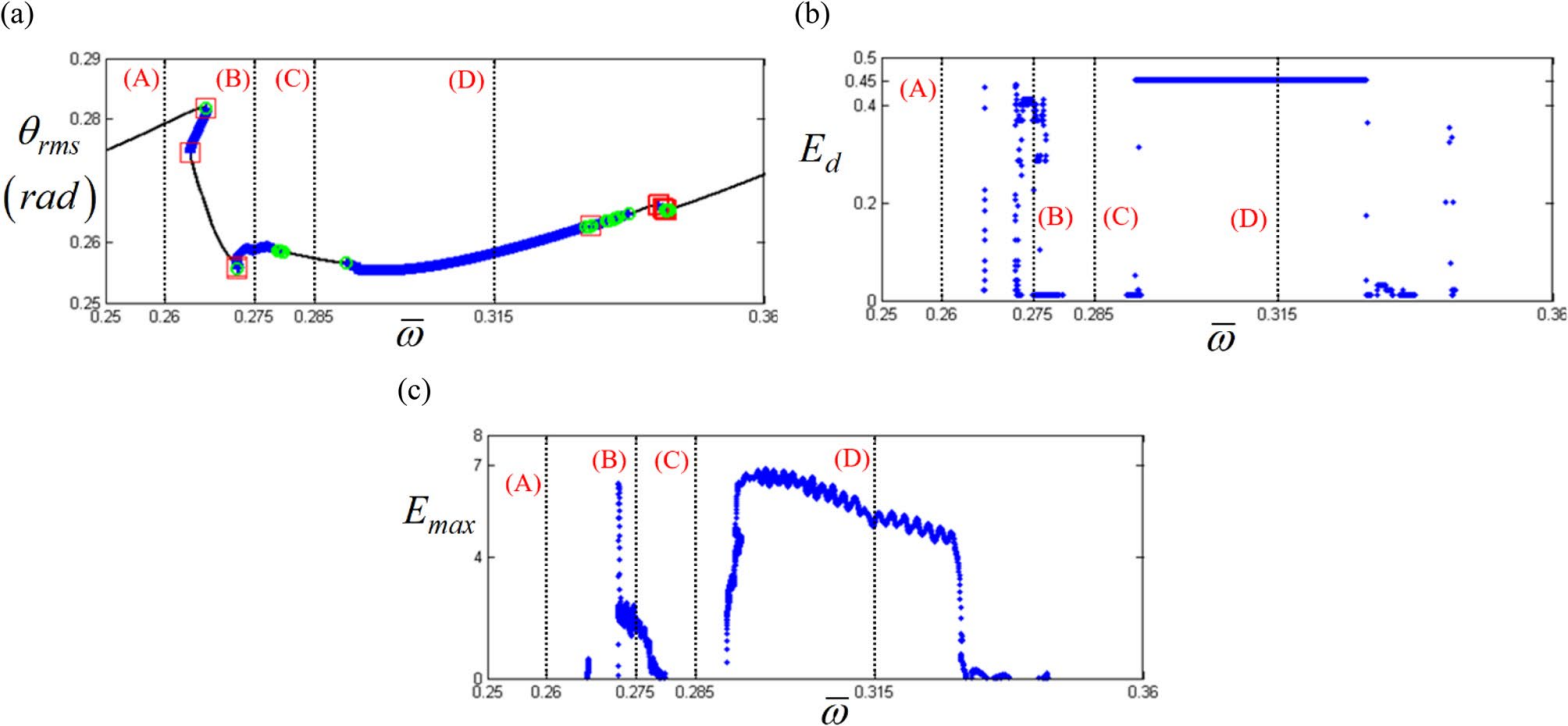
Comparison of bifurcation points with the distribution of eigenvalues at the super-harmonic response regime: (**a**) various bifurcation points; (**b**) ${E}_{d}$ along with various stability conditions; (**c**) ${E}_{Max}$ along with various stability conditions. Key: red open square, saddle-node point; green open circle, bifurcation points other than saddle-node; black line, stable solutions; blue plus, unstable solutions.

**Figure 11:**
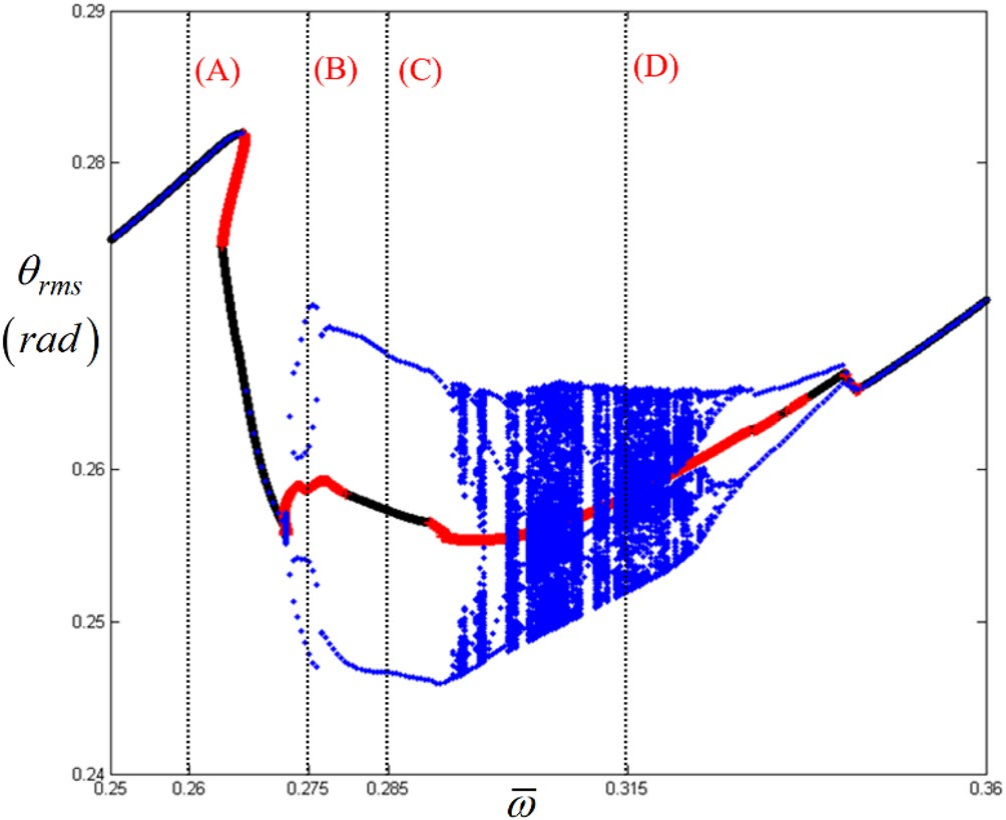
Comparison of HBM and bifurcation diagram with RMS values focused on super-harmonic response regime: Key: black line, HBM result with stable condition; red plus**,** HBM result with unstable condition; blue filled circle, bifurcation diagrams.

Figure 10 compares the HBM results at the super-harmonic resonance with *E*_*d*_ and *E*_*max*_. As marked by (A), (B), (C), and (D), the relevance of *E*_*d*_ and *E*_*max*_ to the bifurcation characteristics is clearly observed. For instance, regimes (A) and (C) do not have any *E*_*d*_ and *E*_*max*_, where any complex bifurcation is expected, as shown in Fig. 11. As shown in Fig. 11, regimes (A) and (C) are related to super-harmonic resonance and period-doubling. However, regimes (B) and (D), where more complex dynamic behaviors are revealed, show effective *E*_*d*_ and *E*_*max*_ values, as shown in Fig. 10b,c. When the range of *E*_*d*_ and *E*_*max*_ values in regimes (B) and (D) are examined, *E*_*d*_ and *E*_*max*_ in regime (D) are higher than those in regime (B). For example, *E*_*d*_ in regime (B) is lower than 0.45, as shown in Fig. 10b. In contrast, *E*_*d*_ in regime (D) is higher than 0.45. Likewise, *E*_*max*_ in regime (B) is lower than 4. However, *E*_*max*_ in regime (D) is higher than 4. When these results are compared to the numerically simulated bifurcations, as shown in Fig. 11, regimes (B) and (D) are affected by the period-doubling cascade and chaotic responses, respectively.

**Figure 12:**
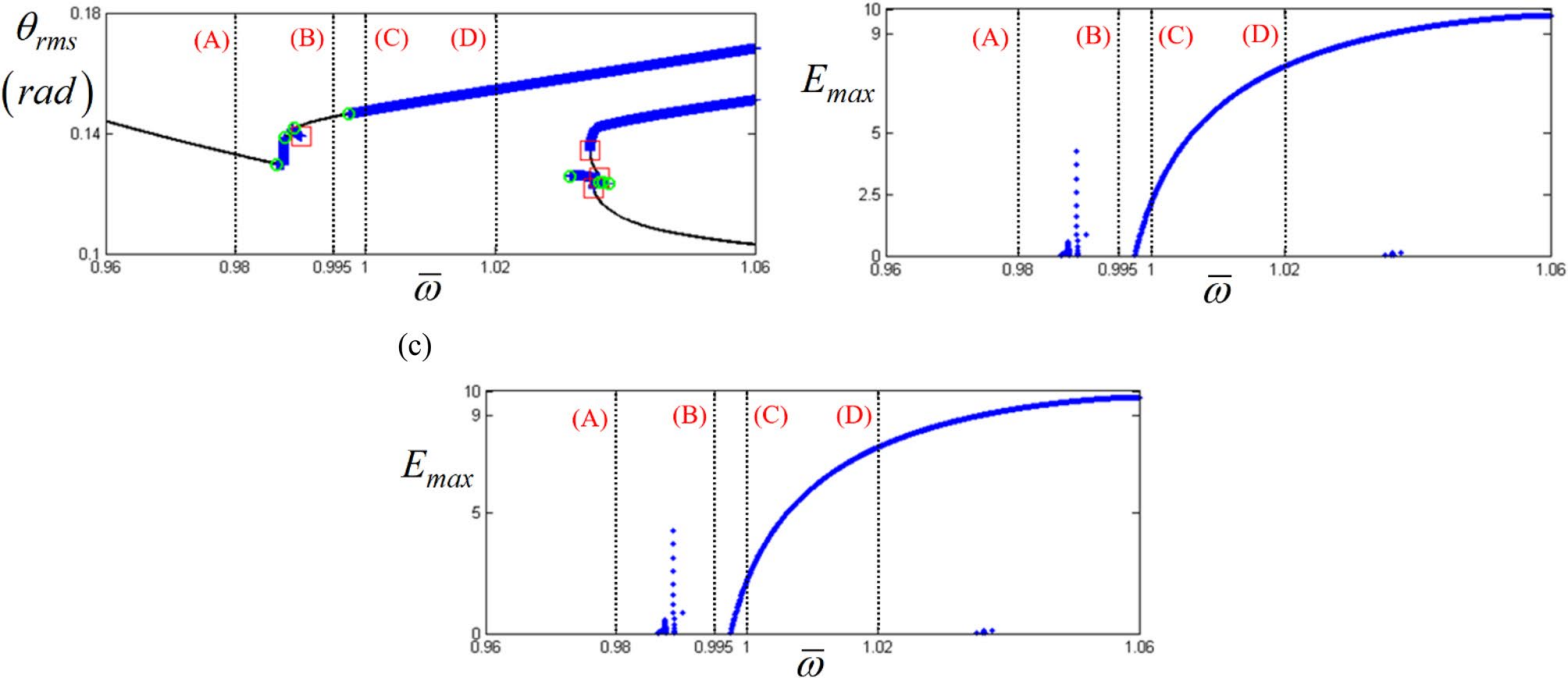
Comparison of bifurcation points with the distribution of eigenvalues at the sub-harmonic response regime: (**a**) various bifurcation points; (**b**) ${E}_{d}$ along with various stability conditions; (**c**) ${E}_{Max}$ along with various stability conditions. Key: red open square, saddle-node point; green open circle, bifurcation points other than saddle-node; black line, stable solutions; blue plus, unstable solutions.

**Figure 13:**
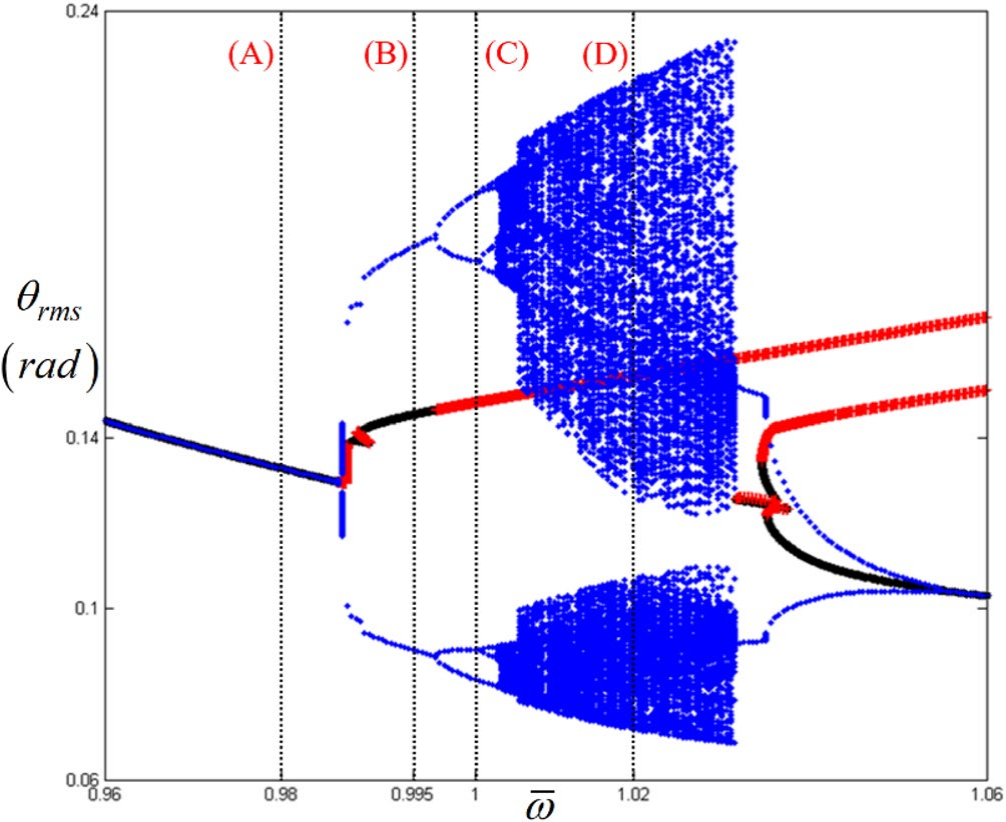
Comparison of HBM and bifurcation diagram with RMS values focused on sub-harmonic response regime: Key: black line, HBM result with stable condition; red plus, HBM result with unstable condition; blue filled circle, bifurcation diagrams.

In addition, Figs. 12 and 13 show a strong relationship between the bifurcation characteristics and the properties of *E*_*d*_ and *E*_*max*_ at sub-harmonic resonance. Here, regimes (A) and (B) do not show any values of *E*_*d*_ and *E*_*max*_, where the sub-harmonic and period-doubling responses are observed, as shown in Fig. 13. However, *E*_*d*_ and *E*_*max*_ in regimes (C) and (D), where more complex dynamic behaviors are observed, show higher values, as shown in Fig. 12b,c. For instance, *E*_*d*_ values in regimes (C) and (D) vary between 4.5 and 5. In addition, while the nonlinear dynamic behaviors are more complex, the level of *E*_*d*_ reaches approximately 0.5. Moreover, *E*_*max*_ increases when the dynamic behaviors change from period-doubling cascade to chaotic response, as shown in Fig. 13.

## Conclusion

This study investigated nonlinear dynamic responses by examining bifurcation characteristics and their relevant properties. In particular, to anticipate complex dynamic behaviors, arc-length vectors and properties of eigenvalues were estimated by employing the HBM. The contributions of this study are as follows: First, this study suggested the numerical techniques to determine BPs on the HBM solutions by estimating the direction of the arc-length vector. From the given results, the BPs were defined as either saddle-node BPs or other types of BPs. Second, the relationships between the bifurcation characteristics and the properties of the eigenvalue real parts, such as *E*_*d*_ and *E*_*max*_, were investigated. Based on these results, while the bifurcation became more complex, *E*_*d*_ and *E*_*max*_ became higher than those in other areas.

There are several possibilities for future research based on the unfinished tasks in this study. For example, the primary resonance has multiple saddle-node points, as shown in Figs. 7a and 8a. In addition, the bifurcation phenomena above $\varpi =1.06$ are still to be analyzed using the results shown in Fig. 4a, which were not fully examined here. These issues will be investigated as the next stage of our research.
